# The Rodin-Ohno hypothesis that two enzyme superfamilies descended from one ancestral gene: an unlikely scenario for the origins of translation that will not be dismissed

**DOI:** 10.1186/1745-6150-9-11

**Published:** 2014-06-14

**Authors:** Charles W Carter, Li Li, Violetta Weinreb, Martha Collier, Katiria Gonzalez-Rivera, Mariel Jimenez-Rodriguez, Ozgün Erdogan, Brian Kuhlman, Xavier Ambroggio, Tishan Williams, S Niranj Chandrasekharan

**Affiliations:** 1 Department of Biochemistry and Biophysics, CB 7260 University of North Carolina at Chapel Hill, Chapel Hill, NC 27599-7260, USA; 2 Present address: Rosetta Design Group LLC, 47 Maple Street Suite 202, Burlington, VT 05401, USA

**Keywords:** Aminoacyl-tRNA synthetases, Urzymes, Genetic code, Origin of Translation, RNA World hypothesis, Amino acid activation, Structural homology, Ancestral genes, Sense/antisense coding

## Abstract

**Background:**

Because amino acid activation is rate-limiting for uncatalyzed protein synthesis, it is a key puzzle in understanding the origin of the genetic code. Two unrelated classes (I and II) of contemporary aminoacyl-tRNA synthetases (aaRS) now translate the code. Observing that codons for the most highly conserved, Class I catalytic peptides, when read in the reverse direction, are very nearly anticodons for Class II defining catalytic peptides, Rodin and Ohno proposed that the two superfamilies descended from opposite strands of the same ancestral gene. This unusual hypothesis languished for a decade, perhaps because it appeared to be unfalsifiable.

**Results:**

The proposed sense/antisense alignment makes important predictions. Fragments that align in antiparallel orientations, and contain the respective active sites, should catalyze the same two reactions catalyzed by contemporary synthetases. Recent experiments confirmed that prediction. Invariant cores from both classes, called Urzymes after Ur = primitive, authentic, plus enzyme and representing ~20% of the contemporary structures, can be expressed and exhibit high, proportionate rate accelerations for both amino-acid activation and tRNA acylation. A major fraction (60%) of the catalytic rate acceleration by contemporary synthetases resides in segments that align sense/antisense. Bioinformatic evidence for sense/antisense ancestry extends to codons specifying the invariant secondary and tertiary structures outside the active sites of the two synthetase classes. Peptides from a designed, 46-residue gene constrained by Rosetta to encode Class I and II ATP binding sites with fully complementary sequences both accelerate amino acid activation by ATP ~400 fold.

**Conclusions:**

Biochemical and bioinformatic results substantially enhance the posterior probability that ancestors of the two synthetase classes arose from opposite strands of the same ancestral gene. The remarkable acceleration by short peptides of the rate-limiting step in uncatalyzed protein synthesis, together with the synergy of synthetase Urzymes and their cognate tRNAs, introduce a new paradigm for the origin of protein catalysts, emphasize the potential relevance of an operational RNA code embedded in the tRNA acceptor stems, and challenge the RNA-World hypothesis.

**Reviewers:**

This article was reviewed by Dr. Paul Schimmel (nominated by Laura Landweber), Dr. Eugene Koonin and Professor David Ardell.

## Open peer review

Reviewed by Dr. Paul Schimmel (nominated by Laura Landweber), Dr. Eugene Koonin and Professor David Ardell. For the full reviews, please go to the Reviewers' Reports section.

## Dedication

“…there is no single path to creativity. We are constrained not by the necessary discipline of rigor but by the limits of our own imaginations and intellectual courage. In the words of Jazz musician Fats Waller, Dare to be wrong or you may never be right.”

- J. Michael Bishop [1]

“How often have I said to you that when you have eliminated the impossible, whatever remains, however improbable, must be the truth?”

- Sir Arthur Conan Doyle [2]

Sergei Rodin (1947-2011) was both a mentor and a collaborator. When the paper that launched this work [3] was challenged [4], Sergei was so incensed that he and his son, Andrei, wrote a brilliant, rebuttal on our behalf [5]. Thus, I also considered him my friend.

The origin of the universal genetic code is one of the most important, fascinating, and vexing questions facing contemporary biologists. Sergei devoted much of his professional life pursuing this question using several perspectives from “outside the box” [5-11]. One of his more unlikely hypotheses was the possible ancestry of Class I and Class II aminoacyl-tRNA synthetases as sense- and antisense- gene products expressed from the same primordial gene [11]. That hypothesis was an elegant realization of thoughts expressed by both Bishop and Doyle. This review considers recent experimental support for, and implications of, their hypothesis.

## Background

**Figure 1:**
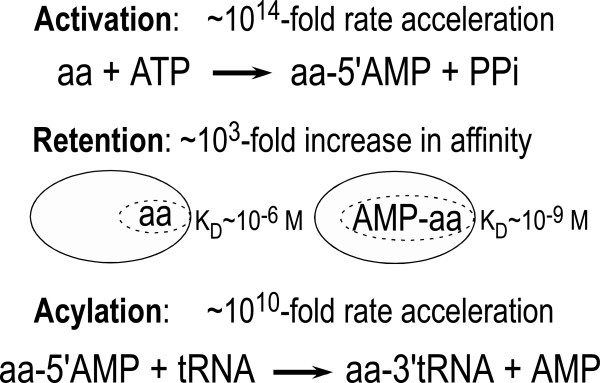
**Aminoacyl-tRNA synthetases have three important functions in the cell.** They use ATP to activate the alpha carboxyl group, making the amino acid more reactive (Activation). This activation step adds the adenosine moiety as an “affinity tag” to the amino acid, increasing its binding affinity by ~1000-fold (Retention) This slows the release of a very reactive species and enables subsequent steps that enhance the fidelity of the final step. Finally, they catalyze transfer of the activated carboxyl group to the 3’ CCA terminus of tRNA (Acylation), completing the translation of the genetic code by the covalent linkage to the tRNA anticodon that is interpreted by the 30S ribosomal subunit in response to codons in mRNA. The approximate rate accelerations achieved by contemporary enzymes indicated are based on comparisons of kcat/K_M_ values to the uncatalyzed rates, estimated from experimental rates of model reactions, as described in [38,39].

The origin of life has challenged scientific investigation for a century or more, generating diverse opinions relating to several disjoint questions about life’s definition in terms of compartmentation, metabolism, and information transfer/storage [12-16]. A central issue linking these questions is a transition from chemistry to biology. Our focus is the origin of codon-dependent translation. Contemporary Aminoacyl-tRNA synthetases (aaRS) accelerate two successive chemical steps: amino acid activation and tRNA acylation. These coupled tasks involve (Figure 1): (i) transduction of the chemical free energy of NTP hydrolysis into the highly unfavorable bond formation leading to mixed anhydride aminoacyl-5’ adenylates, (ii) addition of a covalent tag to the amino acid, i.e., the adenosine moiety, greatly extending the residence time of the activated amino acid within the active site to allow dissociation and/or hydrolysis of incorrectly activated amino acids [17,18], and (iii) specific acylation of tRNA with cognate amino acid. Translation occurs in the third step [19-26].

Absent catalysts, aminoacyl-5’ adenylates form both very slowly and in very low equilibrium yield. The activation step proceeds 10^3^-10^4^ times more slowly than the second in aqueous solution^a^, and therefore requires a correspondingly more potent catalyst. Release and subsequent hydrolysis of the pyrophosphate leaving group are both necessary to ensure that activated amino acids are formed in high yield. Further, once formed, the aminoacyl-5’ adenylate is exceeded in reactivity only by acyl-halides [27]. In fact, of all reactions involved in ribosomal protein synthesis and in both kinetic and thermodynamic terms, amino acid activation is, mechanistically, by far the most challenging.

Just how far back in time the three functions can be traced lies close to the heart of the code’s origins. Consensus holds that aaRS enzymes had essentially assumed their modern configurations in the last universal cellular ancestor, LUCA [28-30]. It is certainly not idle speculation, therefore, that simpler ancestral aaRS preceded LUCA by eons. Nevertheless, because LUCA represents a localized “Big Bang” [31,32], it was associated with intense genetic exchange [33]. Thus, it becomes much more difficult to trace phylogenetic lineages for either activity much beyond that hypothetical landmark. One possible avenue lies in the identification and functional annotation of broadly conserved tertiary packing motifs, illustrated pointedly [34] by a nearly invariant core packing motif belonging to ~ 125 families in the Rossmannoid superfamily. That motif is associated with a discrete supersecondary structure that binds ATP and nucleotides in general, in keeping with its possible role in primordial chemical free energy conversion, and has been identified as a “protoallosteric site” [35]. A related effort is the expression and engineering of invariant cores from enzyme superfamilies [36-40]. We call these constructs Urzymes, from Ur = primitive, original; they are our central focus here (see also [41]).

## Results

### Aminoacyl-tRNA synthetases: why two families?

**Figure 2:**
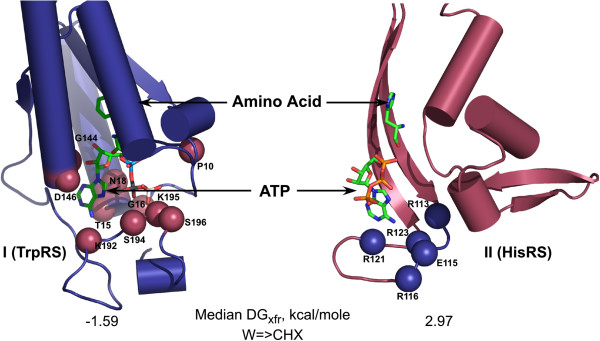
**Detailed comparison between active sites of Class I and Class II aminoacyl-tRNA synthetases.** Substrate binding sites for ATP and amino acid are buried in Class I, and much closer to the surface in Class II enzymes. The color of the active-site spheres illustrates that conserved active-site residues that motivated Rodin and Ohno to advance their sense/antisense coding hypothesis are drawn exclusively from the set of substrates activated by the other class. The median transfer free energy from water to cyclohexane is favorable for Class I substrates and unfavorable for Class II substrates.

The activation and acylation reactions are so important, and the twenty canonical amino acids so diverse, that nature invented two different aaRS families to activate all 20 canonical amino acids. The two enzyme families differ markedly in primary, secondary and tertiary structure [42]. Amino acids activated by Class I and II aaRS divide into apparently symmetrical classes of 10 each^b^, with three comparably sized subclasses, I,IIA(6), I,IIB(2-3), and I,IIC(1-2).Class I and II active sites exhibit several relevant distinctions (Figure 2). First, the amino acid-binding pockets of Class I enzymes lie deep within the Rossmann dinucleotide-binding fold, whereas amino acids bind to a shallow crevice close to the surface of Class II enzymes.

The most highly-conserved aaRS active-site amino acids occur in three sets of signature sequences. We’ll focus on Class I HIGH and KMSKS and Class II Motif 1 and 2. With rare exceptions, conserved amino acids with a direct, catalytic role in Class I active sites are drawn from amino acid substrates activated by Class II enzymes, and conversely (Figure 2). It is hard to imagine how this came about unless the evolutionary ancestors appeared simultaneously, rather than sequentially, as is often argued [43,44].

In any case, sharply contrasting architectures render it inconceivable that the two classes had a common ancestor. Why, then, did nature invent two ways to do the same job? Three related aspects of the class division seem relevant to answering this question:

i. *Coding simplicity*. The earliest proteins probably were encoded by a much simpler genetic code than the code of 20 amino acids we have today. In fact, a binary code of two amino acid *TYPES* that specify “inside” and “outside” seems to represent almost sufficient information (turns excepted) to encode globular objects with selectable functions and hence, to launch natural selection. Combinatorial libraries of polypeptides based on a binary, middle-base code that differentiates only between core and surface amino acids contain high proportions of products with the biophysical characteristics of molten globules [45], and give rise to significant functionalities [46,47]. Two different kinds of synthetases with rudimentary specificities reflected in median hydropathies of the contemporary Class I and II aaRS substrates might thus have been sufficient to launch codon-directed protein synthesis.

ii. *Physical chemistry*. Amino acid substrates of the two classes sort into just such a distinction. The apparent symmetry relating the three subclasses and the inordinate water preference of Class I arginine [48] mask an overwhelming difference between the hydrophobic character of Class IA and Class IIA amino acids. Despite exceptions, Class II amino acids generally prefer the aqueous phase, Class I amino acids the hydrocarbon phase. Their median free energies of transfer of between water and cyclohexane differ by -4.6 kcal/mole [40]. Class I (larger) and Class II (smaller) amino acids are also distinguished by size. Solvent transfer free energy (P < 10^−7^) and mass difference (P < 10^−4^) contribute synergistically (P < 10^−2^) to the solvent accessible surface area in folded proteins (Carter, CW Jr & Wolfenden, R tRNA Acceptor-Stem and Anticodon Bases Form Independent Codes Related to Protein Folding, in preparation); Class II amino acid side chains are, on average, 54% exposed whereas Class I amino acids are 32% exposed (P ~0.03).

iii. *Genetic linkage*. A pre-cellular world populated by quasispecies may have placed a premium on efficient information storage [49]. We further believe that a substantial selective advantage would arise if both required kinds of synthetases were present at the same time and place. Coding Class I and II on opposite strands would link genes for the two classes genetically, assuring that when one was present, so was the other.

### Sense/antisense ancestry

Rodin and Ohno observed a statistically significant complementarity between consensus coding sequences for class-I defining PxxxxHIGH and KMSKS peptide signatures and Class II Motif 2 and Motif 1 sequences, and conversely [11]. They inferred from this that ancestral Class I and II aaRS descended from opposite strands of the same gene, a proposal we call the Rodin-Ohno (RO) hypothesis. Despite the strength of their statistical tests (*vide infra*), it was not obvious when the idea first appeared that experiments could either falsify or confirm this extraordinary proposal. In the interim, however, specifying the hypothesis more precisely in terms of the respective tertiary structures has clarified its more important implications, opening experimental and bioinformatic avenues to assess its validity.

**Figure 3:**
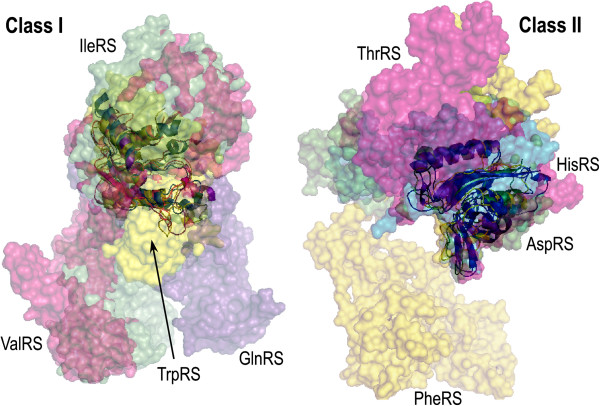
**Superposition of four Class I and four Class II aaRS.** Specific enzymes are colored differently and labeled. Full-length contemporary monomers are shown as surfaces that are 65% transparent, to reveal the invariant cores, shown as cartoons inside the surfaces.

Key to these new developments is the notion that contemporary enzymes grew from ancestral forms similar to invariant cores that can be identified within superimposed members of protein superfamilies (Figure 3). These cores invariably comprise the active sites. They can be expressed in soluble form, sometimes after re-design to modify hydrophobic side chains at newly solvent-exposed surfaces [39]. They exhibit substantial rate accelerations for the reactions catalyzed by the enzymes from which they derive [37-40].

In the following, we first summarize the salient features of Urzymology, the study of Urzymes. Then we describe how Urzymology facilitates the modular deconstruction of Class I TrpRS and Class II HisRS and recapitulation of their evolution. Finally, we summarize how these two Urzymes help validate the Rodin-Ohno hypothesis.

### Urzymology: structural biology yields insights about the invariant cores

Comparative anatomy has always been the *sine qua non* of phylogenic inference. Because our interest here concerns molecules far earlier than LUCA, our approach begins with structural biology and 3D superposition, whose application to aaRS has been reviewed [50]. We used a variety of manual least squares and automated algorithms [51,52] to perform similar analyses. Contemporary aaRS are moderately large enzymes, in keeping with their sophisticated tasks. Their structures also exhibit considerable variation within the two classes (Figure 3). From the outside the four Class I and II monomers superimposed in each part of the figure look quite different. Inside, however, a much smaller, invariant core of ~120-130 amino acids is nearly identical in all 10 examples of each class.

Not surprisingly, cores for each class encompasses the active sites for amino acid activation and acyl-transfer, and hence also the most highly conserved secondary and tertiary structures. Urzymology consists of the methods we have introduced to identify, re-design, express, purify, characterize, authenticate, and exploit the properties of these cores (Table 1).

**Table 1:** Procedures used in Urzymology

**Process**	**Remarks**
Superimpose homologs	POSA server: http://fatcat.burnham.org/POSA/
ID shared invariant subset	Usually 10-30% of monomeric forms
Re-design exposed surface	Protein design: Rosetta http://www.rosettacommons.org/
Clone & Express	Maltose Binding Protein (MBP) fusions; TEV cleavage
Characterize	Rate accelerations, substrate specificities
Authenticate	Single turnover active-site titration, Steady-state kinetics, genetic mutation and manipulation
Evolutionary recapitulation	Multi-scale modular de- and reconstruction

**Figure 4:**
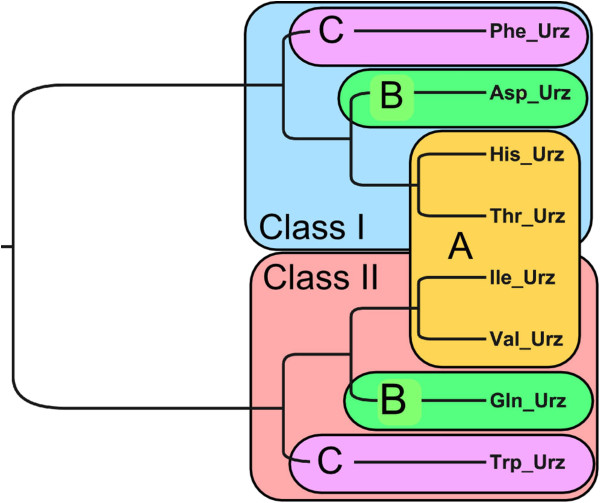
**Partial order structure alignment of four Class I and Class II Urzymes.** Despite the strong similarities between the four Urzymes from each class, POSA identifies appropriate sub-classification.

Whereas invariant cores within each class are very similar to each other, they retain sufficient identity that sub-class relationships are preserved. Four examples (two from subclass A, and one each from subclasses B and C) were superimposed by partial order structure alignment (POSA; http://fatcat.burnham.org/POSA; [52])). Urzymes deduced for members of the same subclass (IA, IIA) were more similar to each other than to members of other subclasses, and the subclasses IC and IIC (Figure 4) were most structurally distinct.

**Figure 5:**
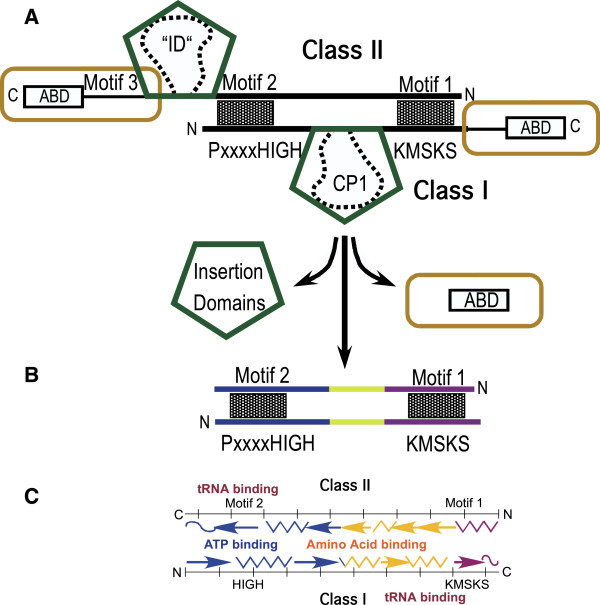
**The Rodin-Ohno hypothesis holds that ancestral forms of Class I and II aminoacyl-tRNA synthetases (aaRS) had fully complementary nucleic acid coding sequences and that contemporary aaRS descended from opposite strands of a single gene.** The schematic in **A** illustrates how this hypothesis leads directly to the concept of aaRS Urzymes. Antiparallel alignment of amino acid sequences for the Class-defining motifs (HIGH and KMSKS from Class I; Motifs 1 and 2 from Class II) reveals that neither anticodon-binding domain, nor a long insertion in each Class can physically be a part of such an ancestral gene. As a result, the only portions of the two Classes consistent with the hypothesis **(B)** are about 120-130 residues long. These fragments coincide with invariant tertiary structural cores shared by all superfamily members. Moreover, these segments together compose a minimal active site, containing binding sites for all three substrates **(C)**. Amino acid and ATP determinants are reflected across the gene sequence, while tRNA binding determinants are related by two-fold rotation [40].

We examine implications from structural biology for the RO hypothesis in Figure 5. Class I and II invariant cores themselves actually can be aligned sense/antisense as noted by Rodin and Ohno [11]. Contemporary aaRS, however, violate this alignment in two ways. (i) Large and variable insertion domains (in Class I these are called Connecting Peptides 1 and 2 [53,54]), interrupt the active-site alignment. (ii) Anticodon-binding domains in both Classes lie outside the range where antiparallel alignment is even possible.

On the other hand, removing all insertion and anticodon-binding domains from both classes leaves the potential sense/antisense alignment intact (Figure 5B). Remarkably, both invariant cores include complete ATP- and amino acid-binding sites, together with rudimentary binding sites for the 3’ CCA termini of tRNA (Figure 5C; [40]).Figure 5C illustrates the chief experimental prediction of the RO hypothesis: parts of either gene that cannot be related sense/antisense—both anticodon-binding domains, the insertions and Class II Motif 3—appear in some sense functionally superfluous. Removing them leaves precisely the invariant cores we had identified for both enzymes, and these align quite closely, sense/antisense. The key experimental question is: how active are Class I and II aaRS Urzymes? Too little catalytic activity to produce activated amino acids at a sufficient rate to support uncatalyzed assembly into peptides would effectively falsify the RO hypothesis. Urzymes are catalytically very much more active than necessary.

### AARS Urzymes both activate, and acylate tRNA with, cognate amino acids

#### Amino acid activation

**Figure 6:**
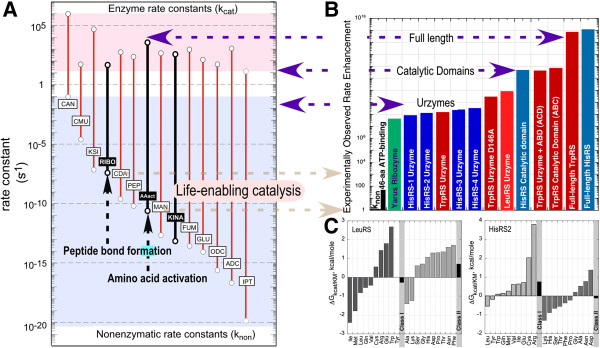
**Quantitative framework in which to assess the catalytic significance of Urzymes and various other putative stages of aaRS evolution. A**. Rate accelerations estimated from experimental data for single substrate (red) and bi-substrate (Black, Bold) reactions adapted from [75] to include uncatalyzed and catalyzed rates of bi-substrate reactions of the ribosome [74], amino acid activation [39] and kinases [106]. Second order rate constants (black bars) were converted into comparable units by multiplying by 0.002 M, which is the ATP concentration used to assay the catalysts shown in **B**. **B**. Experimental rate accelerations estimated from steady state kinetics as kcat/K_M_ for a series of catalysts derived from Class I and Class II aminoacyl-tRNA synthetases ([38,39] and data of V. Weinreb, L. Li, M. Collier, and K. Gonzalez-Rivera presented here in a subsequent section). Vertical scales in A and B are the same, and the origin of the histogram in B has been set equal to the uncatalyzed rate of amino acid activation in (AAact) in A. Red bars denote Class I Tryptophanyl- and Leucyl-tRNA synthetase constructs, blue bars denote Class II Histidyl-tRNA synthetase constructs, and green denotes a ribozymal catalyst [97], included for comparison. Research presented in **A**, **B** was originally published in [37]. © The American Society for Biochemistry and Molecular Biology. **C**. Class I LeuRS and Class II HisRS Urzyme amino acid specificities. Amino acids with more negative ΔGk_cat_/K_M_ values indicate higher activities. By ~1 kcal/mole (light bands) or ~ five-fold, each Urzyme prefers substrates from the class to which it belongs (dark bands). Nonetheless, both activate a range of non-cognate amino acids, and are promiscuous.

Two Class I (TrpRS and LeuRS) and one Class II (HisRS) Urzymes accelerate the rate-limiting amino acid activation reaction ~10^8^-10^9^-fold. These rates are within 10^−5^ (Figure 6; Figure 5 in [37]), so transition-state stabilization free energies of both Class I and II Urzymes are therefore ~60% of, those of full-length aaRS.

#### tRNA aminoacylation

Catalysis of amino acid activation by aaRS Urzymes left a key question unanswered: do these peptide catalysts also accelerate aminoacylation of tRNA? A central implication of the RO hypothesis is that sense/antisense encoded fragments should be exhibit both activities. Precedent and structural arguments led us to expect recognition of tRNA by aaRS Urzymes, even without the anticodon-binding domain, which is often considered a late addition [23]. A considerable literature describes the acylation of isolated tRNA modules containing the acceptor stem [19,20,55,56]. Comparable experiments have until now not been performed with modular fragments of aaRS, owing to the greater difficulty of constructing and purifying proteins, compared to RNA. Further, simultaneous appearance of a fully-developed genetic code, depending heavily on binding the anticodon loop of most tRNAs [56] is difficult to envision. Accordingly, Giegé, Schimmel, and others proposed that an earlier, “operational RNA code” in the tRNA acceptor stem was a forerunner of the present day code [23].

**Figure 7:**
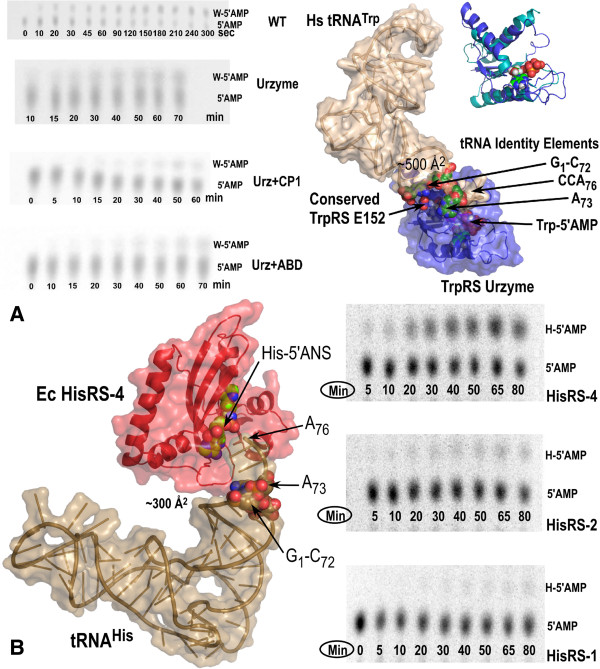
**tRNA**^**Trp **^**Acylation by TrpRS Urzyme. A**. Model of the putative interaction between TrpRS Urzyme and tRNA^Trp^, derived from the crystal structure of the complex between human TrpRS and tRNA^Trp^[57]. Autoradiograms documenting the acylation of tRNA^Trp^ by Wild Type TrpRS, TrpRS Urzyme, and two intermediate modular constructs, containing either CP1 or the anticodon-binding domain. **B**. Model for interaction of tRNA^His^ with HisRS4 Urzyme and autoradiograms showing acylation by HisRS1, 2, and 4 as in **A**. Spheres show Trp-5’AMP, and His-5’ sulfoamyladenylate. These data were published originally in [37] © The American Society for Biochemistry and Molecular Biology.

The crystal structure of human cytosolic TrpRS complexed with the acceptor stem of tRNA^Trp^[57] affords a model for the corresponding interaction with the TrpRS Urzyme (from H. Hu’s MD simulations; [39]) Figure 7A. Buried surface area calculation with a probe radius of 1.5 Å shows that 522 Å^2^ of the Urzyme is potentially in contact with tRNA^Trp^. Further, complementary tRNA-binding surface in the α-helix of the second crossover connection of the Urzyme includes an invariant, E152, that specifically recognizes the discriminator base A73. Similar considerations apply to the HisRS Urzyme•tRNA^His^ complex (Figure 7B).

Data shown in Figure 7A, 7B for ^32^P-3’ adenosine-labeled tRNA^Trp^ and tRNA^His^[58] demonstrate that TrpRS [39] and HisRS [38] Urzymes catalyze tRNA aminoacylation. TrpRS and HisRS Urzymes therefore retain a full functional repertoire. In particular, catalytic activities required for protein synthesis are as finely tuned as the contemporary enzymes, between activation and acylation and between Classes, [37]. They therefore represent convincing models for ancestral Class I and Class II aaRS.

### Are Urzyme activities authentic?

Urzyme catalysis of amino acid activation is ~10^5^-fold weaker than that of contemporary enzymes. How can we be sure that they do not result from a tiny amount–1 in 100,000–of contaminating wild-type enzyme? Seven different tests (Table 2) argue from multiple points of view that these activities are authentic. The last four functional assays uniquely distinguish Urzymes from native aaRS, and are discussed in detail:

**Table 2:** Criteria for the authenticity of Urzyme catalytic activities

**Criterion**	**Implementation**	**Remarks**
Empty vector controls	Purify, assay MBP	*De Rigueur*, but unconvincing
Renaturation from inclusion bodies	Tagged Urzymes purified from pellet	WT Enzymes do not segregate with inclusion bodies
MBP fusions release cryptic activity on TEV cleavage.	Assay fusion proteins ± TEV cleavage	Inhibition in fusion proteins is widespread, not universal.
Active-site titrations Urzyme preparations have significant bursts.	Single turnover time-courses	A key criterion, this is also essential for comparing k_cat_/K_M_.
Mutations, modular alterations induce predictable changes in activity.	Determine effect of active-site mutations, genetic manipulations	Active-site mutations generally affect Urzyme activities differently and can actually enhance activity because mechanisms are different.
Urzymes, WT enzymes have different Steady-state K_M_ values.	Measure: k_cat_, K_M_, k_cat_/K_M_	Contamination by WT enzyme would saturate at WT K_M_.
Amino acid specificity is different from full-length	Compare: (k_cat_/K_M_)_W_/(k_cat_/K_M_)_Y_	Urzymes are generally low specificity, high k_cat_ catalysts.

1. *Empty vector controls show essentially no activity*. We express all Urzymes as maltose-binding protein (MBP) fusions to improve solubility. No unfused MBP expressed and purified in the same manner on an amylose resin exceeded background when assayed at 12 mg/ml with ^32^PPi exchange mixes for tryptophan, histidine, and leucine.

2. *Active TrpRS Urzyme can be renatured from inclusion bodies*[40]. It is unlikely that native full length enzyme would contaminate inclusion body preparations.

3. *Cleavage of MBP fusion proteins releases cryptic activity*. MBP fusions inhibit both TrpRS and HisRS Urzymes ~ 50-fold [38,39]. Cryptic activity released by Tobacco Etch Virus protease cleavage of purified fusion proteins implicates both the purified fragment and protease cleavage (see also 5 below).

4. *Active-site titrations show significant pre-steady state bursts in single turnover assays, amounting to 10-90*% *of the total number of molecules*. Active-site titrations measure single turnover time courses. If product release is rate-limiting, then turnover will be slower than the first round of catalysis, and extrapolation of the steady-state rate to the origin can be used to estimate the “burst” or the amplitude of the first-order portion of the reaction. Burst size therefore estimates the proportion of active molecules. Contaminating activity from a tiny amount of wild type full-length enzyme 10^5^-fold more active than the Urzymes would exhibit an insignificant burst, and the entire time course would represent its steady-state rate. Active fractions also provide more accurate k_cat_ values.

Both Urzymes show substantial bursts, which range between ~10 and 90%—much bigger than those expected from a rare, very active contaminant. Full length aaRS bind tightly to the aminoacyl-adenylate to protect the cell from a highly reactive adenylating reagent and to preserve the specificity achieved by the activation step [17]. It is remarkable that the Urzymes also sequester the intermediate. Pre-steady state bursts are thus a third key function of full-length aaRS (Figure 1) retained by Urzymes from both Classes.

5. *Active-site mutants and modular variants have altered activities*. Molecular biologists recognize that manipulating the gene of a suspected source of activity can implicate that gene product in the observed activity. We therefore tested active-site mutations and modular variants in the TrpRS and HisRS Urzymes. All such experiments significantly altered activity. One result—mutation D146A in TrpRS Urzyme actually increased, rather than reducing activity as it does in the WT enzyme—was counter-intuitive. However, the catalytic function of D146 in full-length TrpRS likely requires allosteric coupling of missing CP1 and ABD modules, and its presence in the Urzyme likely stabilizes the ground state, rather than the transition state [59].

**Figure 8:**
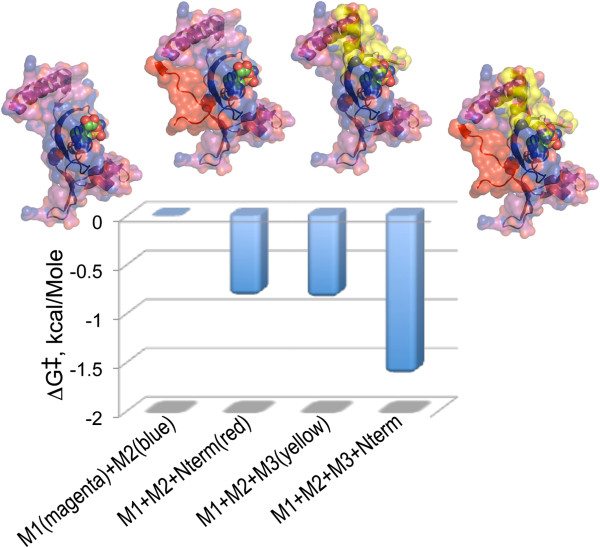
**Quantitative analysis of the catalytic contributions of Motif 3 and a short N-terminal extension of the Motif 1 helix to the acceleration of histidine activation by HisRS Urzyme.** Graphics include the Histidyl-5‘adenylate as spheres. Details of the constructions are described in [38].

Of particular interest are thermodynamic cycles that involve an aaRS Urzyme and complementing segments. Very short (6-20 aa) modules accelerate HisRS Urzyme amino acid activation by a small but significant amount (Figure 8; [38]). We PCR amplified the 122 residue fragment containing only Motifs 1 and 2, adding either a six-residue N-terminal extension (red), or Motif 3 (yellow), or both, giving us a balanced assay for the effects of both factors. The intrinsic catalytic enhancement of Motif 3 to transition-state stabilization by HisRS Urzyme, -0.85 kcal/Mole, is essentially identical to that of the much shorter and less obvious N-terminal extension; their synergistic effect is nearly twice that. Figure 8 emphasizes that the two modules stabilize the ATP binding site from opposite faces of the molecule.

**Figure 9:**
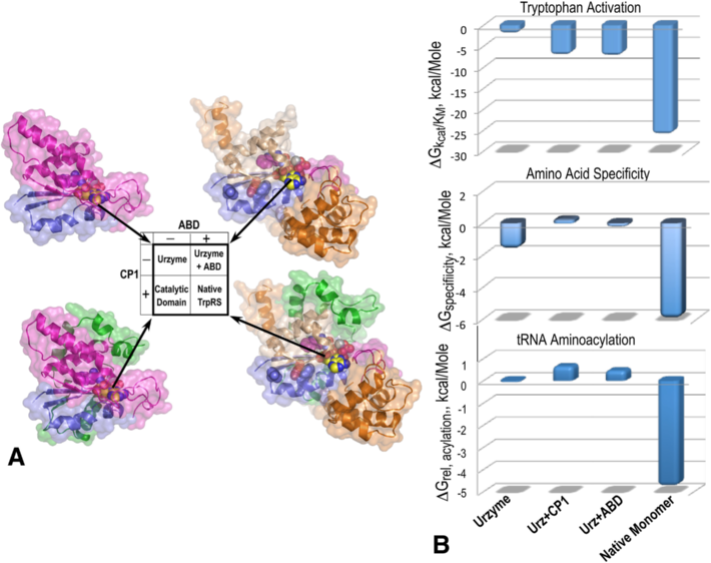
**Factorial analysis of TrpRS inter-domain effects. A**. The catalytic activity of the Urzyme facilitates a full-factorial analysis of the benefits of adding either the CP1 or anticodon-binding domain, jointly with their synergistic effect in the full-length enzyme. **B**. Free energy histograms for the factorial design in **A**, showing nearly identical patterns in which the CP1 and ABD actually diminish both specificity for tryptophan vs tyrosine and acylation of tRNA^Trp^. The entire difference between Urzyme and full-length enzyme is achieved only via the synergistic participation of both domains missing in the Urzyme.

Full TrpRS specificity and tRNA^Trp^ aminoacylation activity both require essentially complete interdomain synergy (Figure 9; [36]). The TrpRS Urzyme favors tryptophan activation by ~10-fold over competing tyrosine and is ~400 times less specific than full-length TrpRS. CP1 and the anticodon-binding domain (ABD) must account for the increased specificity of native TrpRS. We addressed this question by comparing specificities of intermediate constructs in which the CP1 and ABD modules were added back individually to the Urzyme [36] to form a complete factorial experiment in those two variables (Figure 9A).

Quite surprisingly (Figure 9B), although adding back either CP1 or the ABD does enhance tryptophan binding, this effect is non-specific. Addition of either domain also reduced K_M_ for tyrosine, such that the log of the specificity ratio (k_cat_/K_M_)_Trp_/(k_cat_/K_M_)_Tyr_, was actually ~0.0 (Figure 9B). The ~400-fold increase in specificity observed for full-length TrpRS, relative to the Urzyme depends entirely on cooperative interactions (also called epistasis [60-62]) between the two domains [36].

tRNA^Trp^ aminoacylation requires comparable interdomain synergy (Figure 9B). These experiments with TrpRS Urzyme support the unexpected conclusion that the Urzyme itself, consisting only of peptide segments that can align antisense to the HisRS Urzyme, is actually better at the two tasks—amino acid recognition and tRNA aminoacylation—required of aaRS, and hence lie closer to the actual path of aaRS evolution than either intermediate, potentially more advanced construct. Evolutionary development of contemporary enzymes must be more subtle than simply accumulating one module at a time.

6. *Steady state K*_*M*_*values differ from those of the WT enzymes*. Enzymologists recognize that the steady-state K_M_ value is an independent signature. Contaminating wild-type enzymes, irrespective of concentration, would saturate at the same amino acid concentrations. Altered K_M_ values are thus strong evidence against contaminating wild-type enzyme activity. @ ATP- and amino acid-dependent Michaelis-Menten data for the TrpRS and HisRS Urzymes show that ATP binding affinity is either the same or tighter to Urzymes than to contemporary aaRS [38,39]. The k_cat_ values are nearly comparable to those of the native enzymes. Urzyme amino acid K_M_s, however, are quite different from those of full-length, native enzymes. The TrpRS Urzyme K_M_ for tryptophan is ~1 mM, 500 times higher than that of wild type TrpRS [39]. That for HisRS-3, containing Motifs 1, 2, and 3, but lacking the six-amino acid N-terminal extension to Motif 1, is 120 μM, compared to 30 μM for wild-type HisRS and 45 μM for the N_cat_ catalytic domain [38].

7. *HisRS and TrpRS Urzymes have reduced, but Class-dependent specificities*. Weaker amino acid affinities imply that the Urzymes likely have reduced specificity for their cognate amino acids. The TrpRS Urzyme retains a 10-fold preference for tryptophan vs tyrosine [36,39]. Second-order rate constant free energies, -RT ln(k_cat_/K_M_), for amino acid substrates activated by Class I LeuRS and Class II HisRS Urzymes (Figure 6C) show that both Urzymes are promiscuous. However, they both preferentially activate amino acids similar to the original substrate (i.e., Leu and His). By a factor of ~5-fold, they both prefer amino acid substrates from the Class to which they belong. This modest, class-dependent substrate specificity also rules out adventitious activities unrelated to aaRS-derived constructs. As with active-site titration and steady-state kinetic parameters, contaminating wild-type aaRS would have a specificity of ~4000-fold.

Experiments in (1-7) leave little doubt that the Urzymes are the authentic source of observed amino acid activation activities.

### Bioinformatic evidence from multiple sense/antisense alignments

**Figure 10:**
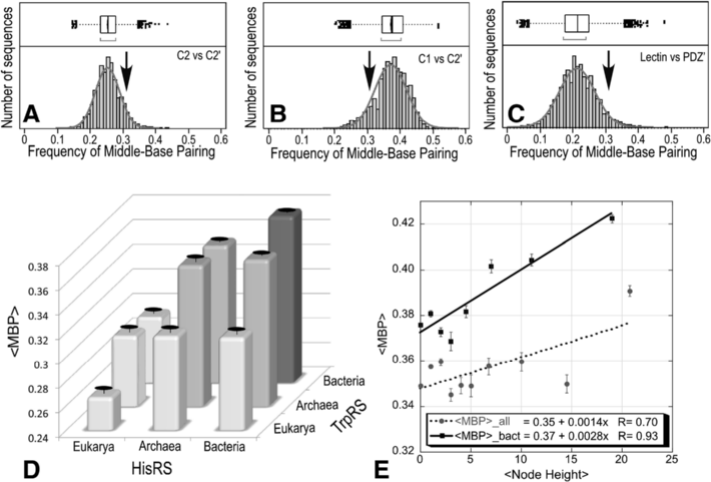
**Evidence for sense/antisense ancestry of the secondary structures connecting catalytic peptides in Class I and Class II aaRS.** Frequency distributions of codon middle base-pairing in control **(A,C)** and antisense alignments of a 94-residue Urgene excerpted from ~200 TrpRS and ~200 HisRS contemporary coding sequences **(B)**. Distributions under the RO hypothesis **(B)** have significantly higher mean values than do those for two samples exhibiting the Null hypothesis that predicts a pairing frequency of 0.25 (one base in four). **D,E**. Domain and evolutionary time-dependent estimates for codon middle-base pairing between antisense alignments of TrpRS and HisRS. **D**. Breakdown of the three consensus domains. The nine columns arise from comparing sequences for one synthetase another when broken down by domain. **E**. Codon middle-base pairing between reconstructed ancestral sequences derived from phylogenetic trees of TrpRS and HisRS Urgene sequences increases as the trees approach the root. Dotted line; all sequences, Solid line bacterial sequences only. (From Chandrasekaran, et al., Mol. Biol. Evol. 2013, 30:1588-1604).

Coding sequences for secondary structure scaffolds that position Class I and II catalytic residues also support Sense/Antisense (SAS) ancestry. Owing to the evolutionary introduction of insertions and deletions, it is significantly more difficult to compare coding sequences for peptides much longer than those analyzed by Rodin and Ohno. Identification and functional validation of the Class I and II Urzymes facilitates extension of coding sequence analysis to neighboring secondary (and tertiary) structures. We described a 94-residue excerpt from the *B. stearothermophilus* TrpRS and E. coli HisRS Urzyme coding sequences [40]. That sense/antisense alignment exhibited conspicuously high (0.44) codon middle base pairing, <MBP>. We generalized that analysis by constructing ~40,000 sense/antisense alignments from ~200 TrpRS and ~200 HisRS sequences distributed throughout the biome [63] (Figure 10B). Codon middle bases pair in 0.34 of all SAS TrpRS/HisRS active-site sequence alignments [63]. This comprehensive analysis sustains our earlier conclusion: the middle base pairing frequencies exceed that observed for various comparable data sets representing the null-hypothesis (Figure 10A,C) that cluster around ~0.25, by a statistically substantial 200-300 times the standard error.

Three additional comparisons between subclasses IC and IIA using sequences for Class IC TyrRS and Class IIA ProRS, with which we rooted the Class I and II phylogenetic trees [63] also all have < MBP > = 0.34 ± 0.002. Middle-base pairing is quite similar in antisense alignments of TrpRS with ProRS, TyrRS with HisRS, or TyrRS with ProRS. Extending this metric to multiple sequences from other subclasses may lead to a deeper phylogeny of aaRS subclasses.

Reconstructed ancestral sequences derived by maximum likelihood methods from the phylogenetic trees show that the middle-base pairing frequencies, which are already markedly higher for bacterial sequences (Figure 10D), also increase for nodes closer to the root (Figure 10E) [63]. Increased ancestral frequencies are consistent with the conclusion that middle codon-base pairing decreases with time, and hence that it was even higher and perhaps equal to 1.0 in the original gene, now inaccessible from the contemporary multiple sequence alignments. Thus, they add weight to the RO hypothesis. Over-represented extant taxa can bias reconstructions [64]. Although middle bases should be least vulnerable to such biases, it is not known how, or by how much, they might affect sense/antisense alignments based on reconstructed sequences from different protein families, and thus how much they strengthen the RO hypothesis.

### An existence proof for embedded modularity and sense/antisense coding

**Figure 11:**
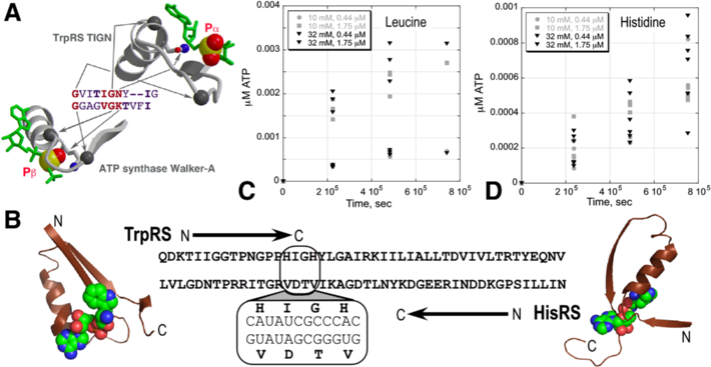
**A *bona fide *sense/antisense gene with amino acid activation activity expressed from both strands. A**. Sequence and structural homology of the Class I HIGH signature and the F1 ATPase Walker A sequence. Glycine residues occur in exactly the same locations. The difference between the two sequences is that the Class I signature is specific for the α-phosphate group, whereas the Walker A signature is specific for the β-phosphate group. **B**. A designed sense/antisense gene coding for the 46-residue Class I aaRS and Class II ATP binding sites on opposite strands. **C**. Time course of leucine activation by the Class I 46mer. **D**. Time course of histidine activation by the Class II 46mer. Both plots have data for two concentrations of amino acid (10, 32 mM) and peptide (0.44, 1.75 μM). Scatter within each set of points is very noisy, so statistical analyses of various dependences are given in Table 3.

We argued previously [37] that the HisRS and TrpRS Urzymes have such finely tuned rate accelerations that they represent highly evolved, relatively recent catalysts, and must have had simpler ancestors. Figure 5C suggests that the substrate binding sites are modular on a scale of tens of amino acids. The N-terminal (TrpRS) and C-terminal 46 (HisRS) amino acids of the Urzymes are ATP-binding modules. The Class I HIGH signature is arguably a version of the Walker-A motif [39] (Figure 11A). Further, a 7-8 residue non-polar packing motif in the C-terminal helix-loop-strand of the N-terminal crossover connection is broadly conserved in Rossmannoid proteins [34]. We have shown the latter motif (the “D1 Switch”) to be central to the allosteric behavior of TrpRS [59,65]. Finally, crystallographic evidence [66] showed that this fragment of TrpRS affords the initial ATP-binding site and roughly half the ATP binding site in the fully closed, Pre-transition state TrpRS conformation.

For these reasons, we characterized the 46-residue peptides from TrpRS and HisRS by several functional assays. Isolated 46-mers from both aaRS classes bind tightly to ATP (KD = 10-65 μM), as has been observed for structurally homologous 45-51 residue fragments isolated from a variety of nucleotide-binding proteins [67-71] containing the Walker-A motif and, by this criterion, may prove to be distant relatives of the Class I aaRS [39].

We (O. Erdogan, B. Kuhlman, X. Ambroggio) set up a direct test of the Rodin-Ohno hypothesis by configuring Rosetta to choose sequences that stabilize the 3D structures of TrpRS and HisRS 46-residue ATP-binding sites, subject to the constraint that the two coding sequences be fully base paired, in keeping with the RO hypothesis (Figure 11B). Catalytic properties of these two gene products have been analyzed extensively (K. Gonzales-Rivera, M. Jimenez-Rodriguez), with attention to likely sources of contaminating activity (Figure 11C,D; Table 3). These experiments approach the background of uncatalyzed rates, and are subject to higher uncertainties. However, Table 3 illustrates that dependences for both peptides on time, amino acid and catalytic peptide concentrations are statistically significant. Similar experiments with purified maltose-binding protein used to solubilize the peptides as a negative control produced an amino acid concentration-independent rate constant of ~4 ± 2 E-12/sec. Rate constants in Table 3 are both ~1.8 E-9/sec and thus accelerate cognate amino acid activation ~400-fold over the uncatalyzed rate, directly validating the RO hypothesis [72].

**Table 3:** Regression analysis of the time, [amino acid], and [catalyst] concentrations of amino acid activation by Class I and II designed sense/antisense ATP binding sites

**Term**	**Estimate**	**Std error**	**t Ratio**	**Prob > |t|**
*CLASS I*				
Time, sec	1.86E-09	1.60E-10	11.62	<.0001
[Peptide], μM	1.50E-03	7.15E-05	20.79	<.0001
[peptide]*[Leucine]	1.46E-05	6.50E-06	2.24	0.0304
Time*[peptide]	9.84E-10	2.45E-10	4.02	0.0002
[Leucine], mM	1.06E-05	4.26E-06	2.5	0.0164
*CLASS II*				
Time, sec	1.76E-09	1.44E-10	12.25	<.0001
[peptide], μM	1.70E-03	7.02E-05	2.43	0.02
[peptide]*[Histidine]	1.43E-05	6.38E-06	2.25	0.03
Time*[Histidine]	2.34E-11	1.31E-11	1.79	0.08
[Histidine], mM	6.31E-06	4.18E-06	1.51	0.14

### Evolutionary enzymology: “life-enabling” catalysis

Arguably, the most important function of enzymes is to equalize reaction rates for the diverse chemistry necessary for life. Catalyzed rates of chemical reactions important for life span at most five orders of magnitude, whereas the corresponding uncatalyzed rates range over 25 orders of magnitude Figure 6A [73-76]. This quantitative framework (Figure 6) lets us assess rate enhancements of the aaRS Urzymes and related catalysts that we have characterized (Figure 6B). Reactions much slower than the fastest uncatalyzed reaction must first be accelerated to about the same rate; otherwise life would be impossible. A key contribution of catalysis, therefore, is to ensure that different kinds of chemistry will happen at close to the same rates.

Assembly of activated amino acids to form peptides is among the faster of the uncatalyzed reactions. Uncatalyzed amino acid activation is three orders of magnitude slower. Uncatalyzed amino acid activation must be accelerated ~1000-fold to provide material for protein synthesis, even in the absence of ribosomes. As described in the previous section, we have found experimentally that catalysts as small as 46 amino acids derived from both aaRS classes afford approximately “life-enabling catalysis” of this essential reaction.

Data in Figure 6C afford a preliminary glimpse at the potential coding properties of the LeuRS and HisRS Urzymes. These are also the first experimental data suggesting that the first proteins were indeed statistical peptides as proposed by Woese [49,77,78] that likely contained sufficient functionality to seed natural selection. We believe that they represent a crude and probably rather late snapshot from the extended process by which decoding proteins (e.g. aaRS) became able to reproduce themselves from self-contained RNA/DNA genes [79]. Additional and more definitive data of this kind, and similar studies of tRNA specificities now afford a new experimental basis from which to unravel that subtle process, e.g. by posing questions such as: can the 46-residue peptide catalysts function using simpler amino acid alphabets?

Further, subsequent evolution from the earliest biological catalysis must have proceeded coordinately, in that reaction rates remained roughly synchronous as their proficiency increased. The histogram in Figure 6B shows experimental catalyzed rates for the aaRS constructs we have made. Class I, II catalytic proficiencies track the structural modularity of the two classes of contemporary aminoacyl-tRNA synthetases, with comparable rate increases over 11 orders of magnitude, providing a crude, but realistic, existence proof of the kind of evolutionary trajectory that led to the contemporary enzymes. Important expeditions forward in time from Urzyme base-camp have now shown that specificity in Class I aaRS requires allosteric behavior in the synergy of two domains, neither of which by themselves enhance fitness [35,36].

## Discussion

### Urzymology has strengthened the posterior probability of the Rodin-Ohno hypothesis

Popper [80] provides an appropriate stance from which to evaluate the RO Hypothesis. It is appropriate to ask whether or not the idea can be falsified by articulating specific predictions derived from the hypothesis, and assessing how new data gathered to test those predictions confirm or invalidate them. Bayes’s Theorem, in turn, provides a quantitative framework for how new data impact confidence in a hypothesis [81]. It asserts that new data update the prior probability of a hypothesis via their conditional probability given the hypothesis, i.e., the likelihood.

Rodin and Ohno adduced a prior probability much higher than generally appreciated. Jumble tests for the observed complementarity relating the conserved Class I and II catalytic signatures had Z-scores ranging from 5.7 to 8.8. Rodin and Ohno understated the corresponding P-values, placing them at “<< 0.01” [11], rather than citing the actual values, 5 × 10^−8^ and 7 × 10^−18^ under the null hypothesis. Sense/antisense ancestry thus begins with a very strong prior probability based on the small statistical chance of otherwise observing the high complementarity between coding sequences for the class-defining motifs.

Predictions of the RO hypothesis have led to new biochemical and bioinformatic data whose probabilities are substantially higher under that hypothesis than under the null hypothesis:

1. *Invariant cores of Class I and II aaRS coincide with the only segments that align sense/antisense*[40]. By consensus, the most conserved amino acid sequences—often catalytic residues at the active site—are the oldest remnants in protein superfamilies. Ancestral gene reconstruction rests on this assumption, whose reliability has been established beyond reasonable doubt for more recent nodes since LUCA with reconstructed nodes defined by large multiple sequence alignments [61,82-89]. Sequence conservation becomes intrinsically less reliable, the further back we reach in time, so structural biology inherits its mantle; we invest conservation of 3D structure with comparable significance.

2. *AARS Urzymes from both classes, solubilized forms of the invariant cores, retain 60% of the transition-state stabilization of contemporary aaRS in both amino acid activation and tRNA aminoacylation, retaining rate accelerations proportional to the respective uncatalyzed rates*[37]. The gap between Urzyme and 46-mer rate accelerations (Figure 6B) is much larger than expected, showing that the most highly conserved secondary and tertiary scaffolds identified using 3D structural alignment are both necessary and sufficient to position conserved active site residues correctly for transition-state stabilization of both amino acid activation and tRNA aminoacylation [36-40]. As the most highly conserved cores actually catalyze the same reactions it becomes increasingly difficult to imagine that ancestors catalyzing both reactions were actually based on non-homologous structures, including ribozymes.

3. *Approximately 70% of the coding sequences (94 of ~130 residues) derived from Class I and II Urzymes exhibit codon middle-base pairing frequencies that are greater by several hundred-fold times the standard error than those expected under the null hypothesis*[63].

4. *Codon middle-base pairing is not significantly different for any of the four combinations between Class IC and Class IIA*[63].

5. *Codon middle-base pairing increases toward the root of TrpRS and HisRS urgene trees*[63].

6. *Highly conserved ATP binding motifs 46 residues long from the Class I and II active sites can be coded by fully complementary nucleic acid sequences, and exhibit 400-fold stimulation over the uncatalyzed rate of amino acid activation (Figure*11*and*[72]). They are phylogenetically and functionally reasonable ancestors of the respective Urzymes.

Probabilities associated with hypothesis testing with Bayes’s Theorem often take the form of odds ratios comparing posterior probability to that of the null hypothesis; the larger that ratio the stronger the case for rejecting the null hypothesis. Alternatively, ignoring the prior probabilities, we can examine likelihood ratios, or how much more probable the new data are under the hypothesis to be tested than under the null hypothesis. For large numbers, the logarithm of the likelihood ratio or log-likelihood gain affords the relative “support” [90].

**Figure 12:**
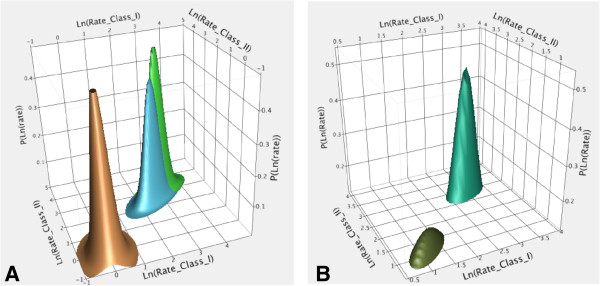
**Illustrative use of Bayes’s Theorem to estimate the enhancement of posterior probability under the RO hypothesis given by the catalytic activities of the 46-residue ATP binding sites expressed from a designed, sense/antisense gene.** All probability distribution functions are bivariate normal probability distributions. **A**. Prior probabilities centered at a mean rate acceleration of 1000 (green), and for the null hypothesis (brown) that the peptides are catalytically inactive. The likelihood function derived from experimental results obtained for Class I and II ATP binding sites (blue) is centered at a rate acceleration of 400-fold. Standard deviations of all three distributions in A are 0.4 log_10_ units, corresponding to a 40% uncertainty. X and Y axes are logarithmic in the rate enhancement, and 1000-fold is drawn from the ratio of the uncatalyzed rates of activated amino acid assembly to form peptides to that for amino acid activation by ATP. **B**. Posterior probabilities obtained for the null prior (purple) and for the two active peptides (turquoise). The integrated experimental posterior probability under the RO hypothesis is larger than that under the Null hypothesis by ~10^14^. The latter posterior probability (brown) has been multiplied by a factor of 10^14^ to be visible.

Although items 1-6 above all have intuitively very large log-likelihood gains, few are readily calculated. An illustrative exception is the 400-fold rate acceleration for amino acid activation by the 46-residue peptides expressed from both strands of the designed sense/antisense gene. In this case, we can estimate the odds ratio of posterior probabilities that both peptides are sufficiently active catalysts to launch ribosome-independent peptide synthesis under the RO and null hypotheses (Figure 12). The non-uniform prior for the RO hypothesis is a bi-variate normal distribution—one dimension for each class—centered on the ratio of uncatalyzed rates for peptide synthesis from activated amino acids and amino acid activation, or log(1000) (Figure 12A). The prior probability for the null hypothesis (i.e., peptides from both classes are catalytically inert) is a similar bi-variate normal centered at 0. The blue surface is the likelihood function for results in Figure 11. Posterior distributions are products of the two priors by the likelihood (Figure 12B). That for the RO hypothesis has an integral ~10^14^ times higher than that under the null hypothesis. Urzyme-catalyzed rate accelerations are 4-5 orders of magnitude greater than expected. Their posterior probabilities are correspondingly smaller, establishing the 46-residue peptides, not the Urzymes, as the most credible models for the catalysts necessary to launch peptide synthesis.

### The RNA World hypothesis

*Copernicus famously said that Earth revolves around the sun. But opposition to this revolutionary idea didn’t come just from the religious authorities. Evidence favored a different cosmology*[91]*.*

“People may spend their whole lives climbing the ladder of success only to find, once they reach the top, that the ladder is leaning against the wrong wall” - Thomas Merton

The genetic code is undoubtedly the nexus between chemical evolution, where genetic inheritance is meaningless, and biological evolution, from which we can in principle trace phylogenies. Conventional wisdom holds that this nexus was traversed exclusively by RNA molecules. That hypothesis is broadly held to be the only likely scenario for simultaneous introduction of genetic information and catalysis [15]. Belief in the early importance of RNA-only metabolism continues to be strong and actively pursued [92], to the exclusion of alternatives. Sergei Rodin was a persuasive and imaginative advocate of this notion [6].

There is broad consensus, which we share, that RNA was a carrier of information mediating the origin of codon-dependent translation. Catalysis, however, is an entirely different matter. The RNA World hypothesis rests almost exclusively on engineering and selection of RNA aptamers capable of RNA replication [93,94], amino acid recognition [95], aa-5‘AMP synthesis [96], and tRNA aminoacylation [97]. The relevance of such experiments is hard to assess. They arise because oligonucleotide syntheses are so accessible, and because SELEX technology can select from extraordinarily large combinatorial libraries. Indeed, given such awesome selective power, it would be surprising not to have identified such aptamers. Without genetic evidence linking them to biology it is difficult to attach significance to their catalytic activities.

Before our work on aaRS Urzymes, the prior [98], alternative case for a peptide/RNA origin rested on three arguments. (i) Modeling suggests that even the emergence of RNA and the establishment of the code required the catalytic repertoire of stereochemically complementary polypeptide hairpins. (ii) RNA in biology is made entirely by proteins and conversely, proteins are assembled by ribozymes, suggesting that this may always have been the case. (iii) The complete absence of contemporary catalytic RNA genes for either ribozyme catalyzed free-energy conversion (amino acid activation by ATP) or codon-dependent translation (specific recognition and catalysis of acyl-transfer to tRNA) argues persuasively that these processes may never have been catalyzed by ribozymes. The magnitude of this gap in phylogenetic support is substantial and, in our view, decisive.

The unexpected catalytic power of relatively simple peptides allows us to invest the peptide/RNA alternative scenario with a much higher probability [37,98,99], in part because it implements rudimentary sense/antisense stereochemical coding of two amino acids per base of an RNA double helix. The unexpected sophistication of aaRS Urzymes implies that they had even simpler ancestors. The two aaRS classes are certainly among the oldest, if not the oldest, protein superfamilies. The RO hypothesis [11] implies that they arose at nearly the same instant in geological time because, at the nucleic acid level, the information necessary for function of each Class is indistinguishable from that necessary for function of the other. Complementarity means that one implies the existence of the other. Sense/antisense coding thus projects back past the genetic coding nexus to chemistry.

By greatly reducing the information necessary to launch natural selection, Urzymes strengthen the case that it arose from a balanced peptide-RNA partnership. The origins of natural selection are rooted in catalysis—producing some molecules faster than others. As amino acid activation rates limit spontaneous peptide synthesis, the initial selective advantage of the earliest catalysts was probably the ability of ancestral synthetases to mobilize ATP (NTPs) and to activate amino acids, enhancing rates at which peptides could be made. In this scenario, information and catalysis both began simply and evolved together. Rather than an unaccountable burst of both information and catalysis, the peptide/RNA scenario lays out a credible path to complexity.

The transition from chemical evolution to genetic biology nevertheless remains baffling. Questions remain about how Urzyme coding sequences began to function as genes. However, at some point, that did happen. Codon middle-base pairing frequencies establish a phylogenetic lineage that projects peptide-mediated catalysis further toward the origin of life than was previously considered possible, to events at the origin of translation in a peptide/RNA world [37].

## Conclusions

The RO hypothesis is certainly falsifiable [80]; three orthogonal but equally rigorous tests fail to do so. Invariant cores that align sense/antisense have considerable and comprehensive catalytic activities. Coding sequences for 70% of Class I, II Urzymes exhibit unexpectedly high middle-base pairing. Products from a designed, sense/antisense gene for Class I and II ATP binding sites both exhibit appropriate catalytic activities. Our formulation is Popperian [80] and Bayesian [81]: these data have very high probability under the RO hypothesis, which is thus a much more probable explanation than others of how the two aaRS Classes arose.

Urzymes demonstrate that the most highly conserved segments, by themselves, have high activity but low specificity—the very properties expected of catalysts implementing the genetic code. By Ockham’s razor, the true ancestral aaRS were unlikely to have differed greatly from those of the TrpRS and HisRS Urzymes. More generally, Urzymes are authentic catalysts that model very early enzymes. Their enzymatic activities provide valid metrics for testing improvements in fitness from modules as small as 6-20 aa [38] and for novel thermodynamic cycle analysis of contemporary enzyme function [36,35].

## Methods

Aspects reviewed in this work were carried out using methods described in detail in the original publications [36-40,63]. Briefly, invariant cores from both aaRS superfamilies were identified by 3D superposition [52], re-designed if necessary using Rosetta Design [100], expressed either with FLAG and His_6_ tags or as maltose-binding protein fusions, purified using these tags, and assayed as described [40].

All statistical calculations were performed using JMP [101]. P values under the null hypothesis in the description of amino acid physical chemistry were estimated from multivariate linear regression models in which the dependent variable (i.e., solvent accessible surface area of each amino acid in folded proteins) is expressed as a linear combination of other predictors, amino acid mass and solvent transfer free energies for each amino acid, plus their interaction.

Data presented in Figure 6C were obtained by Michaelis-Menten steady-state kinetics as in [36] in which all amino acids (excepting tyrosine because of its limited solubility) were substituted individually for cognate amino acids. Four-fold replicate assays were performed on two occasions and all replicated data were treated independently in nonlinear fits to the Michaelis-Menten equation using JMP. Maximum velocities were divided by the concentration of active sites to give k_cat_ values. Proficiencies, k_cat_/K_M_, were converted to free energies and plotted.

ATP was titrated at pH = 4.5 with increasing amounts of 46-residue segments isolated from TrpRS and HisRS by PCR amplification and purified by affinity chromatography as noted in the following paragraph. This assay, described by Mildvan [102] detects fluorescence changes as the peptide orders and binds to ATP. ATP affinities were estimated from the titration curves using JMP [101]. TrpRS and HisRS 46-residue peptides were also assayed by ^32^P PPi exchange, essentially as noted in the next paragraph.

Data presented in Figure 11 were obtained as follows: Rosetta was adapted (OE, XA, BK) to constrain sequences simultaneously for two backbones provided as scaffolds with the additional constraint that substitutions at each position have complementary codons, assuring that the resulting gene was fully sense/antisense. The resulting genes were inserted separately in opposite directions for expression as MBP fusion proteins, expressed and purified by affinity chromatography on amylose, nickel-NTA, and blue sepharose supports and stored in 50% glycerol at -20 C. Time-dependent assays were sampled at 0, 3, 6, 9, and 12 days in parallel with background controls using the standard ^32^P PPi exchange assay conditions [40]. After determining that tryptophan over such long incubations induces elution from charcoal of a yellow compound that independently enhances scintillation counting, leucine was used to assay the Class I ATP binding site. Histidine did not show such behavior and was used in the assay of the Class II ATP binding site. Additional controls were done separately using maltose-binding protein itself with both amino acids, as isolated from amylose chromatography.

## Reviewers’ reports

### Reviewer 1: Dr. Paul Schimmel, *Skaggs* Institute for Chemical Biology at The Scripps. Research Institute (nominated by Prof Laura Landweber)

This review recapitulates the long-standing Rodin-Ohno hypothesis that, by postulating that complementary strands of early genes encoded members of the two classes of tRNA synthetases, the mystery of two classes is solved. Specifically, this complementarity is proposed to come from the active-site-encoding mRNA of one synthetase (say, from class I) being the anti-sense of the active-site-encoding mRNA of a synthetase from the opposite class (say, class II). Thus, one duplex encodes both types of synthetases. In the active-site encoding region, there is a group of ‘codons’ in the strand encoding a class I synthetase that are paired (in the duplex gene) with the corresponding group of codons in the strand encoding a class II tRNA synthetase. In their original work, RO presented evidence from the existing sequence databases to support their hypothesis. These databases have grown enormously since then and have provided further opportunity to test this hypothesis.

Carter, in a virtually single-handed way, has attempted to dig more deeply into the predictions of the hypothesis through experiments and bioinformatics. His first paper in Molecular Cell (Carter and Duax (2002)) was a provocative discovery of how the complementary strands of the NAD-GDH gene, in the fresh water mold *Achlya klebsiana*, code for the two different class-associated synthetase signature motifs. Thus, these gene-encoded signature motifs are in exact complementary alignment with each other in the *A. klebsiana* NAD-GDH gene. (Surprisingly, this paper is not cited.) This remarkable finding gave Carter the impetus to search out experimental proofs of the RO hypothesis, using peptide motifs that embodied the signatures of the class I and class II synthetases, and testing their abilities to stimulate amino acid activation. It also stimulated him to dig more deeply into the bioinformatics.

This review is a compendium of much of that work. The paper recapitulates the experiments of his laboratory, and also summarizes some deeper bioinformatics. The experimental work is outlined in great detail. An impressive long summary is given of experiments to prove that the results are not artifacts. And yet, by giving this long list, this summary has the appearance of being defensive. There also are ‘bumps’ , such as the failure to obtain clear results with the ‘mutants’ of the peptide motifs. And there is always the philosophical problem of ‘the absence of evidence of an artifact is not evidence of absence’. Although the work described is well done and rigorously thought through, Carter et al’s strong enthusiasm for their work on the peptides gives a sense of bias.

The section entitled “Bioinformatics evidence from multiple sense/antisense alignments” impressed me. Carter has great strength in this sort of analysis and presents an excellent update and extension of the earlier RO work. Likewise, the section of the Discussion entitled “Urzymology has…the RO hypothesis” is an excellent point-by-point of the state of affairs on the informatics side. I was quite impressed by the depth of this recapitulation.He views the work as providing a challenge to the RNA World hypothesis. I found this viewpoint somewhat curious. For myself, the two lines of thinking can by harmonized in a straightforward way, by an extension of the idea first described in Figure 2 of a rather obscure publication (Henderson, B. S. and Schimmel, P. (1997). RNA-RNA Interactions Between Oligonucleotide Substrates for Aminoacylation. Bioorgan. Med. Chem. 5: 1071-1079.) In that publication, the authors suggest that ribozymes first aminoacylated small RNAs, and then these aminoacyl RNAs formed clusters that brought together the activated amino acids to form peptides. I can imagine some of these peptides eventually associated with the ribozymes and enhanced their catalytic activities. I would also imagine that these peptides could be related in some way to the ones that Carter and his students have so well studied. Whether or not these ideas are correct, my main point is that Carter et al. have nothing to gain by attempting to provide a contrast with the RNA World hypothesis. Their work stands well on its own merits.

Overall the review is written in a somewhat rambling, diffuse style, and with emotional content. (I noted 5 exclamation points scattered throughout the text, and recommend that these be removed to give a lack of bias and to convey “academic sobriety”).

I am a fan of Carter and his work. He is widely respected for his thorough and deep understanding of thermodynamics and protein structure-function. His experiments are generally thorough and self-critical. He is the only person who not only expanded the analytical side of informatics that is relevant to the RO hypothesis, but also has done specific experiments to test the hypothesis. This sort of combined effort is rare in any field. My recommendation is that the paper be published. It is a fascinating topic that Carter alone is in the best position to summarize. But the text needs to be tightened up, shortened, and recast in a more sober style, considering some of the points raised above.

**Authors’ response**: *We appreciate both the complimentary assessment of the work overall and the criticism of the writing. We readily eliminated all exclamation marks. It takes nothing away from the text to suppress hyperbolae. We have three substantive replies:*

***The extant *** **A. klebsiana *sense/antisense gene.*** *Professor Schimmel asks why our first publication on the RO hypothesis was not cited. In fact, it was cited implicitly in the dedication to Sergei Rodin. That section now includes explicit references both to our paper (Carter & Duax, 2002) and to the paper challenging aspects of the work (Williams, et al., 2008), as well as the rebuttal published by Rodin, Rodin, & Carter (2009). Carter & Duax based their work on that of H. LeJohn, in which he used antibody precipitation to clone a stress-induced glutamate dehydrogenase*[103-105]*. The cloned gene expressed both the putative dehydrogenase and HSP70. Although LeJohn’s work was described in some detail in the three resulting papers, Williams, et al. (2008) concluded that the evidence that the protein expressed from the putative dehydrogenase clone was indeed the dehydrogenase was weak, and hence that the interpretation reported in Carter & Duax might be flawed. We decided against re-opening this issue here, in part because it contributes little to the subsequent work reviewed here on aaRS Urzymes, and because we have done little to resolve discussions with Dr. Koonin about a related issue concerning possible ancestral relationships of the Class II aaRS, actin-HSP70, and RNAse H superfamilies.*

***Mutational analysis of Urzymes and 46-mers.*** *Professor Schimmel writes “There also are ‘bumps’, such as the failure to obtain clear results with the ‘mutants’ of the peptide motifs.” His reference fails to distinguish between two possibilities: (i) the D146A active site mutation in the TrpRS Urzyme actually increases activity and (ii) we have yet to obtain similar results for the 46-mer SAS gene products. Regarding (i), we can now postulate and are testing a coherent explanation for the unexpected activation of the TrpRS Urzyme by the D146A mutation. This explanation is now outlined in the appropriate section of “Are the Urzyme activities authentic?” point 5. Regarding (ii), we are in the process of testing active-site mutants to both 46-mers. The activities of these peptides are more difficult to validate than those of the Urzymes, owing to the fact that it is unlikely that they retain activated aminoacyl-adenylates and their active site titers cannot be determined as they were for the Urzymes. The authors feel that data in Table*3*afford compelling, though admittedly not definitive evidence of authenticity and hence justify publication of the data in Figure*11*C,D.*

***The RNA World hypothesis.*** *Professor Schimmel questions our assessment of the RNA World hypothesis, suggesting that the essential validity of the RO hypothesis is neither evidence for nor against that scenario, and arguing that hypothetical RNA and peptide/RNA scenarios can be reconciled along lines he developed in an earlier paper. We disagree substantively on both points. The paper he cites documents an intriguing model for peptide synthesis from acylated RNA stems. However, it fails to address the fundamental issue posed by the RNA World hypothesis: where did RNA arise if not via rudimentary catalysis by peptides? The absence of present day ribozymes related in any phylogenetic sense to the any of required activities of the aminoacyl-tRNA synthetases or indeed to nucleic acid polymerases, should be a massive red flag. The sense/antisense ancestry of the aaRS appears to be solidly established. It points, intrinsically, far further back in time than do multiple sequence alignments for any gene, establishing phylogenetic roots of the earliest coded peptides.*

The RNA World hypothesis suppresses entire domains of important questions related to the physical chemistry of proteins and catalysis, including the absence of phylogenetic evidence for ribozymal nucleic acid polymerases. On the other hand, the contributing author’s alternative scenario, published a dozen years before Gilbert’s proposal, suggests coherent answers to many of these questions, and in addition affords a rudimentary, but consistent, path backward to a putative earlier sense/antisense genetic coding. Thus, there is much for both the authors and the literate scientific public to gain by revisiting that alternative hypothesis, especially as we are the ones who have resurrected it from oblivion with key catalytic activities that establish its credibility as an alternative. Suppressing discussion can hardly be productive.

Professor Schimmel’s discomfort with our discussion of competing hypotheses is, however, likely exacerbated by the imbalance of substance and polemic in that particular section of the submitted manuscript. We have re-balanced this section by removing >25% of the text, most of which was either polemical or redundant, and by supplementing it with additional references on ribozymal aptamers. Elsewhere, the manuscript has been tightened throughout and is now ~5% shorter overall despite the inclusion of new material responding to these and other reviewers’ comments.

### Reviewer 2: Dr. Eugene Koonin, National Center for Biotechnology, NIH

This very lengthy, yet carefully and elegantly written article summarizes experimental data from the senior author's laboratory that is perceived to support the Rodin-Ohno hypothesis on the origin of the two classes of aminoacyl-tRNA synthetases from complementary strands of the same gene. I expect this paper to become an important contribution to the literature on the origin of codon-dependent translation. It contains a plethora of interesting ideas and descriptions of ingenious experiments. After quite some thought, I have decided not to comment in specific detail on the Rodin-Ohno hypothesis and the validity of the presented argument in that regard. Again, Carter and colleagues present their argument in detail and with great care, so an interested and qualified reader will be able to judge it.

**Authors’ response**: *We are grateful to Dr. Koonin both for his generous remarks and for having provided in Biology Direct an appropriate venue in which to generate public dialog of topics growing from the work described. As noted above, the revision benefits from tightening and some re-structuring.*

### Reviewer 3: Professor David Ardell, University of California, Merced

This work by Carter et al. reviews a substantial and growing literature on testing and extending the Rodin-Ohno hypothesis using ‘urzymes’—experimentally tractable models of early aaRSs. It presents new data on the substrate specificities of urzymes—and describes a designed experimental ‘existence proof’ of complementary antisense coding of Class I-type and Class II-type amino acid activation activities.

The Rodin-Ohno hypothesis is consistent with persuasive ideas about early life. For instance, in their original work, Rodin and Ohno speculate that sense-antisense coding may, via compression, bring replication advantages to quasispecies. Overlapping genes may also be favored through co-transfer of co-dependent ‘decoding genes’ during code evolution in structured populations (Vetsigian et al. (2006) in “Collective evolution and the genetic code” PNAS 103(28):10696).

The present work by Carter et al. places weight on Rodin and Ohno’s own statistical analyses using permutation tests: ‘jumbling’ as according to R. Doolittle. By design, these jumbles are not constrained to conserve sequence, particularly the critical active site motifs of the two classes of aaRS. Should we not instead attempt to model the space of all possible sequences with primordial Class I-type and Class II-type aaRS activities, and use this as a condition when measuring the probability of complementarity? What indeed are the spaces of all shortest protein sequences with Class I-type or Class II-type enzymatic activities comparable to (those of) urzymes? Or to frame the question differently, how ‘designable’ are the Class I and Class II aaRS active sites (‘Sequence optimization and designability of enzyme active sites’ by Chakrabarti et al. (2005) PNAS 102(34):12035)? Were the four motifs—HIGH, KMSKS, Motifs I and II and the extended secondary structures studied by the authors of the present work—inevitable products of selection for these activities? If the motifs and structures were inevitable, then perhaps their encoded head-to-tail antisense complementarity is just a remarkable coincidence.Of the new data presented by the authors, I found the data in Figure 6C of interest, reporting activities of urzymes in activating different amino acids in ambiguous, yet apparently class-specific, ways. These data have not been published elsewhere, and I caution that I was unable to evaluate them critically. At face value they raise the question whether the ‘statistical urzymes’ implied by them have class-specific amino acid activation activities or not. It would seem to strengthen the Rodin-Ohno hypothesis if they did, but not necessarily refute it if they did not. A refinement of this question would take into consideration that not all amino acids were available in early ancestral genetic codes. Generally speaking, at what rates could antisense-coded urzymes regenerate themselves in model prebiotic translation systems? For a relevant theoretical treatment of this question, please see Bedian (2001). “Self-description and the origin of the genetic code”. BioSystems 60:39.

Part of the authors’ case in this review rests on ancestral reconstructions of aaRS coding sequences. These reconstructions must be among the most ambitious possible, in terms of the depth of reconstruction and base composition nonstationarity of the data being modeled over the Tree of Life. Nonstationarity is especially problematic in causing bias in ancestral sequence reconstructions (Susko and Roger (2013). “Problems With Estimation of Ancestral Frequencies Under Stationary Models”. Syst Biol 62:330). I don’t believe that the results discussed in this work, on complementarity in reconstructed ancestors, adequately controls for this bias.The following statement “Without exception, conserved amino acids with a direct, catalytic role in Class I active sites are drawn from amino acid substrates activated by Class II enzymes, and conversely” seems misleadingly strong to me given data from single structures shown in Figure 2. This is a much stronger claim than Rodin and Ohno themselves made in their analysis of sequence variation (on page 568 of their work).

The statistical methods leading to the p-values reported in the Physical Chemistry section on page 8 should be briefly summarized.

**Authors’ response**: *The authors are especially grateful for the careful reading and thoughtful criticism from Dr. Ardell, who identified several intriguing questions, some that we feel largely lie outside the scope of this article, but which point directly toward future investigations. We were unaware of several references provided and have tried to cite them appropriately in the revision.*

***Self description and the origin of the genetic code.*** *We appreciate the identification of previously unpublished work presented in two sections, the specificity spectra of Class I TrpRS and Class II HisRs Urzymes, and characterization of the sense/antisense gene for the 46-residue ATP binding sites of Class I and II synthetases. In order to facilitate more critical assessment, we have amplified the description in Methods of how specificities for each amino acid were determined. As with much of what is reviewed in this paper, in this these data represent the first attempt to bring the underlying question raised by Professor Ardell further in par 4 of his review into an experimental context. These preliminary data are certainly neither comprehensive nor definitive. Nonetheless, they represent an honest attempt to provide experimental bases from which, eventually, to approach the question posed by Bedian. We agree wholeheartedly with Professor Ardell that the aminoacyl-tRNA synthetases lie at the center of the genesis of biological self-reference represented by the genetic code. Our work thus far has helped only to define experimental systems with which this problem can be fruitfully addressed. Our revision includes a new paragraph just before the Discussion in which we touch briefly on the questions posed by Bedian. Our work is still at the beginning of the effort to answer the question; thus it seemed inappropriate to speculate further.*

***Designability****. This is an excellent question, one about which we have thought quite a lot. In an as yet unpublished study of the LeuRS Urzyme construction, we characterized eight different Urzymes designed by Rosetta. These Urzymes had a narrow range of specific activities. That is, they all had almost the same activity as the re-designed TrpRS Urzyme. That Rosetta design experiment does not fully address Professor Ardell’s question, however, because we did not allow changes to active-site residues; nor did we constrain the design of catalytic hydrogen-bonding interactions. We note here, however, that this question is in some ways simply a re-phrasing of the question addressed in the preceding paragraph. Curiously, however, more in-depth comparison now reveals functionally important differences between some of the LeuRS Urzymes we selected for further work. Notably, variation in the loop connecting the specificity determining helix to the GXDQ motive at the N-terminus of the C-terminal alpha helix generates two LeuRS Urzymes, one of which exhibits a pre-steady state burst, the other of which does not. Pursuit of that question is obviously a valid future research project.*

***Non-stationarity of ancestral character states****. This is also an excellent question. We were unaware of the potential bias identified in the paper by Susko and Roger, which appeared online only in September 2013, by which time our own paper had been published for two months. We certainly will attempt to take the bias into account in future work. We have qualified our interpretation of the reconstructed sequences in the revision. The middle-base pairing frequency statistic, which as Professor Ardell correctly states is among the most ambitious metrics ever proposed for phylogenetic comparisons, has independent validity irrespective of whether or not we reconstruct ancestral states. The data in Figure*10*B and*10*D reflect frequencies from contemporary sequences, not reconstructed ancestral states.*

***Interdependence of Class I, Class II aaRS****. Professor Ardell questioned the strength of the phrase “Without exception..” in referring to the active-site compositions in Class I aaRS, which are constructed from Class II substrates, and vice versa. We have qualified the statement in the revision. Figure*2*is indeed drawn based on only a single Class I active site and a single Class II site. However, a comprehensive examination of active site compositions across the ten members of each family from several hundred species does reinforce that description. The HIGH signature contributes three residues H, G, and H that interact with ATP. G is essentially invariant because it is in van der Waals contact with the adenosine ring, whereas the two Hs are replaced only by T and N, and for example never by Q, which is in other contexts a functional substitute for both H and N. Similarly in the KMSKS motif the four catalytic residues are always K, S, and T. The only exceptions we know of are from eukaryotic TrpRSs and TyrRSs in which the terminal lysine is absent (replaced by A) and its function is taken by an arginine that occurs uniquely in these enzymes much closer to the amino terminus. Similarly all 10 Class II active sites invariably use R for transition state stabilization. The active site E is only very rarely a D. Thus, the statement discussing Figure*2*is scarcely hyperbole. The distinction between our treatment and that of Rodin and Ohno is that the latter authors included nonpolar amino acids that couple the active site residues to the rest of the protein, whereas we consider only those residues that interact directly with ATP.*

***Statistical methods****. It is fair to ask for clarification of how P-values were calculated. This is explained more fully in a new addition to the Methods section.*

## Endnotes

^a^Estimated rates of uncatalyzed reactions are summarized in [38,39].

^b^Noncanonical Class I lysyl-, Class II Pyrrolysyl-, and variations of non-discriminating Class I glutamyl and Class II Aspartyl-tRNA synthetases lie outside the scope of this review [24].

## Abbreviations

aaRS: Aminoacyl-tRNA synthetases; LUCA: Last universal cellular/common ancestor; CAN: Carbonic anhydrase; CMU: Chorismate mutase; KSI: Ketosteroid isomerase; RIBO: Ribosome; CDA: Cytidine deaminase; PEP: Carboxypeptidase; AAact: Amino acid activation; MAN: Mandelate Racemase; KINA: Kinases; FUM: Fumarase; GLU: Sweet potato β-amylase; ODC: Orotidylate decarboxylase; ADC: Arginine decarboxylase; IPT: Inositol phosphatase; AST: Alkylsulfatase.

## Competing interests

None of the authors has any competing interests.

## Authors’ contributions

CWCjr: conceived and directed all projects in his own laboratory, and wrote the manuscript. VW: devised the assay used to increase sensitivity of the ^32^PPi exchange assay, characterized the activities of the 46-mer peptides, critically reviewed and revised the manuscript. LL: constructed and characterized the HisRS1-4 Urzymes and carried out tRNA acylation assays, measured the specificity spectrum of HisRS 2, critically reviewed and revised the manuscript. OE: designed and MLC expressed and characterized the LeuRS Urzyme, critically reviewed and revised the manuscript. OE, BK, and XA: adapted Rosetta for the design of, and designed the sense/antisense 46-mer gene, critically reviewed and revised the manuscript. KG-R: characterized the activities of the 46-mer peptides, critically reviewed and revised the manuscript. MJ-R: characterized the activities of the designed sense/antisense 46-mer peptides, critically reviewed and revised the manuscript. TW: characterized the activities of the 46-mer peptides, critically reviewed and revised the manuscript. SNC: produced the bioinformatics data and deduced that the sense/antisense relationship relating Class I and II signatures extended to the secondary structural scaffolds, critically reviewed and revised the manuscript. All authors read and approved the final manuscript.

## Authors’ information

This review originated in a presentation by CWCjr at a memorial symposium celebrating scientific contributions of Sergei Rodin, (1947-2011) at the Beckman Center, City of Hope, Duarte, CA. It is dedicated to the memory of Rodin and Ohno. None of the authors has a conflict of interest.
